# Revisiting how we perform late gadolinium enhancement CMR: insights gleaned over 25 years of clinical practice

**DOI:** 10.1186/s12968-023-00925-0

**Published:** 2023-03-16

**Authors:** Elizabeth R. Jenista, David C. Wendell, Clerio F. Azevedo, Igor Klem, Robert M. Judd, Raymond J. Kim, Han W. Kim

**Affiliations:** 1 Duke Cardiovascular Magnetic Resonance Center, DUMC-3934, Durham, NC 27710 USA; 2 Division of Cardiology, Department of Medicine, Duke University Medical Center, Durham, NC 27710 USA; 3 Department of Radiology, Duke University Medical Center, Durham, NC 27710 USA

## Introduction

Late gadolinium enhancement (LGE) imaging has become one of the cornerstones of the core cardiovascular magnetic resonance (CMR) examination [1]. Also known as delayed enhancement imaging, LGE enables the differentiation of viable from non-viable myocardium in a wide range of patients with ischemic and non-ischemic myocardial disease and is now considered the imaging reference standard for the diagnosis of myocardial infarction and scarring.

With the increased adoption of CMR by the cardiovascular community, LGE is being more frequently performed in patients with significant comorbidities, such as acute heart failure and arrhythmias. In these patients, optimization of acquisition parameters can significantly improve image quality. Moreover, new LGE techniques, including dark blood delayed enhancement, have been described and appear to have additive clinical utility. Thus, it is timely to present an update on how we perform LGE at our center [2]. We will review the sequence variants used, key imaging parameters, as well as the adjustments that can be made to optimize image quality, and highlight potential pitfalls that we have encountered in our experience using this method over the last 25 years. We will also end with a brief discussion of image interpretation focused on the assessment of myocardial viability in patients with chronic ischemic heart disease.

### Protocol

LGE imaging is a fundamental component of the core CMR examination. The inclusion of LGE imaging allows the simultaneous visualization of both infarcted and normal myocardium. At our center, LGE imaging is performed in the vast majority (> 98%) of studies. LGE images are typically acquired after cine imaging and other morphologic imaging or mapping. When vasodilator stress perfusion imaging is also performed, LGE is typically obtained after rest perfusion imaging. When vasodilator stress perfusion imaging is also performed, LGE is used to both differentiate infarcted from ischemic myocardium and to distinguish true ischemic perfusion defects from perfusion imaging artifacts.

#### Contrast dose and type

**Fig. 1:**
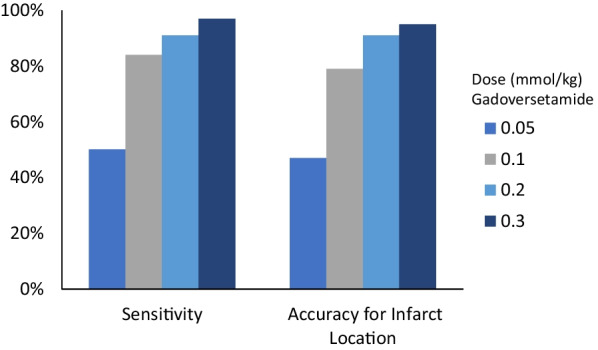
The relationship between the diagnostic performance of late gadolinium enhancement (LGE) and gadoversetamide contrast dose. Adapted from Kim et al. [3]

We typically administer 0.15 mmol/kg of gadolinium contrast media for viability imaging alone or 0.2 mmol/kg in two divided doses during vasodilator stress and rest perfusion imaging. Lower doses of contrast media can be considered for viability imaging, however, this may reduce the diagnostic performance of the LGE imaging, particularly at 1.5 T [3]. The relationship between LGE diagnostic accuracy and contrast dose was demonstrated in an international multicenter study which evaluated the performance of LGE for the detection of myocardial infarction (MI) (Fig. 1). This study found a significant dose–response relationship (both P < 0.0001) of gadoversetamide with sensitivity and accuracy for infarction location increasing with rising contrast dose [3].

There are several factors that merit consideration when selecting a gadolinium-based contrast agent (GBCA) for LGE imaging. At our center, we use group II GBCAs due to their favorable safety profiles with few (if any) associated cases of nephrogenic systemic fibrosis [4]. We also find that the rate of contrast agent clearance from the blood pool is an important consideration when choosing a contrast agent for LGE. For example, albumin binding GBCAs provide relatively persistent and high blood pool enhancement, often for more than 10–20 min following contrast administration. Although helpful for contrast enhanced CMR angiography, the long duration of blood pool enhancement with albumin binding GBCAs can be problematic for LGE imaging since the blood pool signal and adjacent subendocardial LGE (if present) will have similar (bright) image intensities, potentially leading to difficulty in consistently identifying subendocardial MI. At the other end of the spectrum, we have found that some GBCAs exhibit very rapid clearance from the body. The latter can be problematic (especially for less experienced operators) because there is a relatively short time window during which LGE images with optimal image contrast can be acquired. In instances for which image quality is insufficient, a small additional dose of contrast can be considered.

#### LGE sequence variants and image timing

Our imaging protocol for LGE utilizes the following pulse sequence variants for most patients:

1. Bright blood 2D free breathing single shot inversion recovery (SSIR) balanced steady state free precession (bSSFP)
2. Bright blood segmented 2D breath held inversion recovery (IR) fast gradient echo (GRE) or segmented 2D breath held flow independent dark blood delayed enhancement (FIDDLE).

**Fig. 2:**
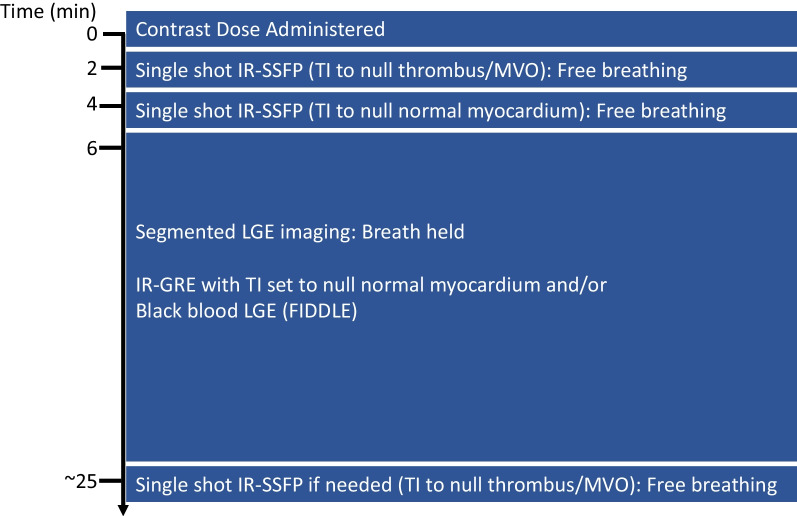
Timeline and LGE pulse sequence variants used at our center. IR-bSSFP: Inversion-recovery balanced steady state free precession, IR-GRE: Inversion recovery gradient echo; TI: Inversion Time; MVO: Microvascular obstruction

We typically begin SSIR-bSSFP image acquisition within 2–3 min of contrast administration (Fig. 2). Image stacks are prescribed over the entire heart in the short axis and in the LV 2-chamber orientation. These images are acquired free breathing with a high inversion time (TI; ~ 600 ms at 1.5 T and ~ 875 ms at 3 T), which nulls thrombus or regions of myocardium with microvascular obstruction (MVO). Next, the imaging stacks are repeated in both orientations with a TI set to null normal myocardium. Third, breatheld segmented IR-GRE and/or FIDDLE images are acquired in the left ventricular (LV) short and long axis views (2-, 3-, and 4- chamber views). In patients in whom mural thrombus or MVO is initially suspected, the SSIR-bSSFP images with the long TI can be repeated in order to help differentiate between the two tissue types (see Inversion Time Section for more details).

We do not use a standard or fixed wait time (e.g., ~ 8–10 min after contrast administration) before starting LGE imaging for two reasons. First, intracardiac thrombi and regions of myocardium with MVO are visible immediately after contrast administration on SSIR-bSSFP images with a high TI. Second, when attempting to visualize infarction or scar, there is substantial patient to patient variation in the clearance of contrast, and using a fixed wait time before acquiring LGE images can result in missing the optimal imaging window in individuals with rapid contrast clearance. Given these considerations, we begin high TI LGE imaging shortly after contrast administration.

These different pulse sequence variants for LGE imaging can be complementary within the same patient. With SSIR-bSSFP, image acquisition is fast and does not require breath holding, permitting a rapid survey of the heart. With the segmented sequences, spatial and temporal resolutions are higher than for SSIR-bSSFP, however, breath holding is required to eliminate image ghosts due to respiratory motion. For segmented IR-GRE, the higher resolution leads to improved sensitivity for the detection of scar and better visualization of MI transmurality as compared to the single shot variant [5].

**Fig. 3:**
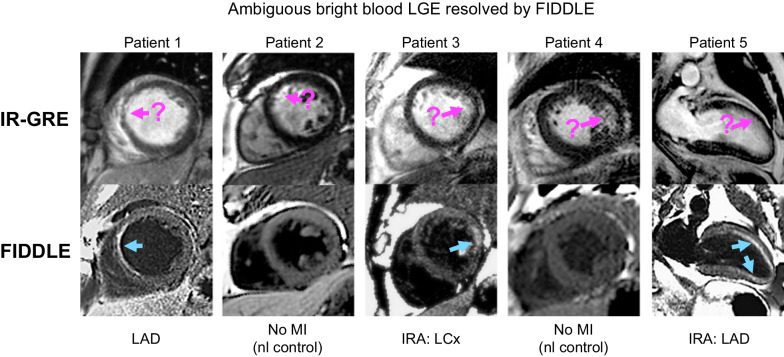
Images in 5 patients (n = 3 with myocardial infarction (MI), n = 2 control patients without MI) in whom the diagnosis of MI was ambiguous on conventional bright blood LGE (IR-GRE). In patients 1 and 2, there is possibly anteroseptal wall hyperenhancement on IR-GRE. FIDDLE shows unequivocal LGE in patient 5, but also demonstrates no LGE in patient 6, who is a normal control. Similarly, in patients 3 and 4, there are ambiguous regions of LGE on the IR-GRE images involving the lateral wall. FIDDLE identifies that patient 3 has lateral wall infarct, while patient 4 is a normal control. In patient 5, FIDDLE demonstrates not only a subendocardial infarct in the anterior wall but also extension into the inferoapical wall. IRA: infarct-related artery; IR-GRE: inversion recovery gradient echo; LAD: left anterior descending; LCx: left circumflex; MI: myocardial infarction; RCA: right coronary artery. Adapted from Kim et al. [6]

Over the last ~ 15 years, we have added the FIDDLE imaging protocol. This is because dark blood LGE techniques further improve the identification of myocardium with contrast enhancement [6]. In particular, with standard bright blood LGE techniques (SSIR-bSSFP or IR-GRE), the hyperintense signal intensity of the LV cavity, caused by the presence of intravascular contrast material, reduces the contrast-to-noise ratio between blood and infarcted myocardium and hampers clear infarct delineation. In patients with subendocardial MI or scarring, this is often problematic since the infarct can be indistinguishable from the adjacent blood pool, potentially leading to a missed or erroneous diagnosis (Fig. 3). In patients, FIDDLE has been shown to have improved diagnostic performance for the diagnosis of MI as compared to conventional bright blood IR-GRE (sensitivity: 96% vs 85%, respectively, p = 0.002; accuracy: 95% vs 87%, p = 0 0.01) [6].

An alternative to segmented breatheld bright blood IR-GRE is the acquisition of multiple SSIR-bSSFP images that are subsequently motion corrected, co-registered, and averaged [7]. The technique produces images that have hybrid characteristics of the SSIR-bSSFP and segmented IR-GRE. Similar to an individual SSIR-bSSFP image acquisition, ghosting artifacts due to respiratory motion are not present, while the spatial and temporal resolutions are comparable to that of segmented IR-GRE through the application of parallel imaging. The signal-to-noise ratio (SNR) loss associated with parallel imaging is offset through averaging of the images (typically 8 averages, acquired over 16 heart beats). In the LV short axis orientation, motion correction is able to account for variations in the in-plane position of the heart, and the sequence provides image quality that is similar to that segmented IR-GRE, but without the requirement for breath holding. However, in the LV long axis views, image co-registration can be inaccurate due to the greater through-plane motion of the heart associated with free breathing, potentially producing artifacts within the composite image. Nonetheless, the technique is very helpful in patients who are unable to comply with breath holding instructions.

### Pulse sequence parameters

In this section, we will discuss the parameters of the bright blood and dark blood pulse sequences used at our center and describe how we train operators to make patient specific adjustments.

#### Bright blood SSIR-bSSFP and segmented IR-GRE

**Fig. 4:**
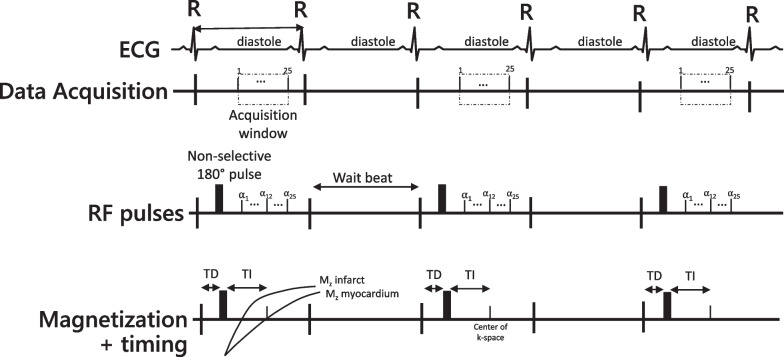
Timing diagram of two-dimensional segmented inversion-recovery fast gradient echo pulse sequence. ECG: The ECG tracing is used to trigger the image acquisition in mid-late diastole. Data Acquisition: The dashed box indicates the time during which the data is acquired (“Acquisition window”) which is controlled by the repetition time and the number of lines of k-space being acquired. RF pulses: The sequence begins with a non-selective 180° inversion pulse, followed by the readout pulses. The subsequent heart beat is a “wait beat” to allow for magnetization recovery between inversion pulses. Magnetization: After the detection of the R-wave, there is a time delay (TD) before the inversion pulse is played. The inversion pulse inverts the magnetization, which recovers based on the tissue T1. The recovery curves for infarct and myocardium are shown to highlight the differences in magnetization recovery due to the differences in post-contrast T1. At the time where the center of k-space is acquired, the magnetization of the myocardium is at the zero crossing, while the infarct magnetization is above the zero crossing. The resulting image contrast is that the myocardium has very little signal (magnetization is near zero) while the infarct is bright (significantly above zero). ECG: electrocardiogram. RF: radiofrequency; TD: trigger delay; TI: inversion time delay; α: shallow flip angle excitation

Typical pulse sequence parameters at 1.5 T and 3 T for the SSIR-bSSFP and segmented IR-GRE implementations used at our center are shown in Table 1 and 2. LGE images are obtained in short axis views (~ 7 mm slice thickness, 3 mm gap) every 10 mm from the mitral valve insertion to the LV apex, as well as in 3 dedicated long axis view 4-chamber, 3-chamber, and 2-chamber). The timing diagram for segmented IR-GRE is shown in Fig. 4. As technical details of the pulse sequences have been described elsewhere [2, 8–10], we will focus upon the key parameters that our scan operators commonly adjust in order to improve image quality.

**Table 1:** Typical parameters for 2D IR-GRE

Parameter	1.5 T	3.0 T
FOV	300–380 mm	300–380 mm
In-plane voxel size	1.2–1.5 × 1.5–2.0 mm	1.0–1.3 × 1.3–1.7 mm
Matrix size	256 × 194	288 × 230
Slice thickness	7 mm	7 mm
Flip angle	20–30°	15–20°
Segments	13–31	25–40
Inversion time (TI)	Variable	Variable
Bandwidth	130 Hz/pixel	347 Hz/pixel
TE (echo time)	3.38 ms	2.27 ms
TR (repetition time)	8.8 ms	5.03 ms
Gating factor	2	2
K-space ordering	Linear	Linear
Fat Sat	No	No
Asymmetric echo	Yes	Yes
Grad moment refocusing	No	No
Parallel imaging factor	None	2

#### Data acquisition window

The time during which the image K-space data are acquired by the CMR scanner is the data acquisition window (Fig. 4). For both SSIR-bSSFP and segmented IR-GRE implementations, the scan operator may adjust the location and/or the duration of the acquisition window with the goal of limiting the impact of cardiac motion on image quality.

**Fig. 5:**
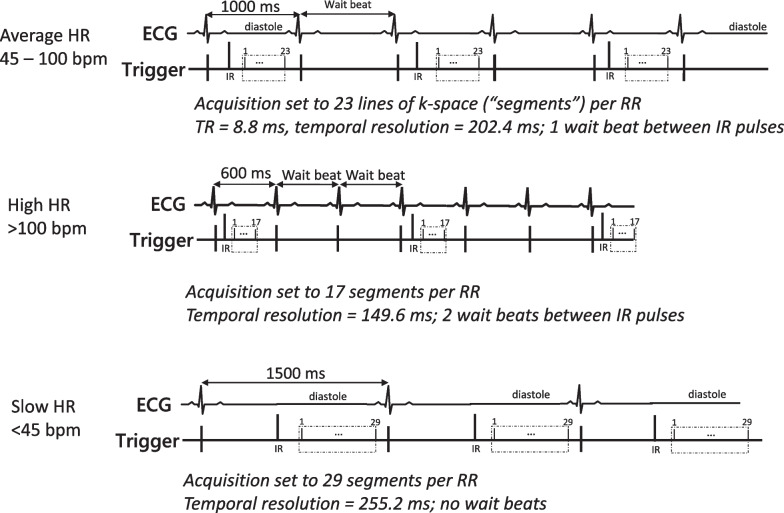
Data acquisition duration and gating factor depending on heart rate. TOP: ECG and readout timings for patients with typical heart rates seen in clinical practice (~ 45–100 bpm). Inversion pulses are played every other heartbeat to allow for longitudinal magnetization recovery between inversion pulses. MIDDLE: In patients with tachycardia (> 100 bpm), two parameter adjustments may be considered. First, the duration of the readout may be reduced by lowering the number of segments (lines per segment), improving the temporal resolution. Second, two wait beats (gating factor of 3) may be used, which allows greater recovery of longitudinal magnetization between inversion pulses. BOTTOM: In patients with bradycardia (< 45 bpm), a gating factor of 1 (no wait beats) can be considered. The time between inversion pulses is greater with very slow heart rates. Accordingly, there is sufficient recovery of longitudinal magnetization with no wait beats between inversion pulses. The duration of the acquisition window may be modestly increased to account for the longer prior of the diastolic standstill period

The location of the acquisition window is adjusted to occur in mid-diastole of the cardiac cycle when the heart is relatively motionless. Typically, the acquisition window is ~ 200 ms in duration in patients with heart rates between ~ 45 and 100 bpm (Fig. 5). The end of the acquisition window corresponds to the end of the diastolic standstill period and usually occurs prior to the P-wave of the electrocardiogram (ECG) tracing. If there is ambiguity when LGE imaging should be performed (e.g., due to dys-synchronous contraction associated with left bundle branch block), operators can use cine imaging to identify the standstill location and duration more precisely.

The duration of the acquisition window is the temporal resolution for LGE imaging and is determined by the number of K-space lines (segments or view per segment) per heart beat. There are several factors to consider when adjusting the duration of the acquisition window depending upon the sequence used.

For segmented implementations, the acquisition of K-space occurs incrementally over several heart beats. The number of phase encoding lines acquired in each heart beat determines temporal resolution (number of phase encoding lines multiplied by repetition time; e.g., 23 lines × 8.8 ms = 202.4 ms). When fewer lines are acquired per heart beat, the temporal resolution is shorter but the total number of heart beats (and breath hold time) to acquire the data needed for reconstruction is higher (matrix size divided by segments).

For single shot implementations, all the K-space lines for an image are acquired in a single heart beat. The temporal resolution is determined by the total number of K-space lines acquired in phase-encoding direction and the parallel imaging acceleration factor. For any given matrix size and acceleration factor, the number of K-space lines acquired alters the temporal resolution (e.g., more lines, increases readout duration). As such, single shot implementations are typically limited by worse spatial resolution for the same temporal resolution as compared to segmented implementations.

Altering the duration of the acquisition window or temporal resolution may be advantageous in specific instances for segmented K-space implementations (Fig. 5). For example, in patients with significant tachycardia (~ > 100 bpm), reducing the number of segments may improve image sharpness since temporal resolution is improved (shorter; e.g., 17 segments × 8.8 ms = 149.6 ms). Conversely, increasing the number of segments (e.g., 29 segments × 8.8 ms = 255 ms) may provide acceptable temporal resolution with minimal change in image sharpness when the heart rate is slow (~ < 45 bpm) since the diastolic standstill period is longer. The latter change carries the additional benefit of reducing the overall breath hold time.

**Fig. 6:**
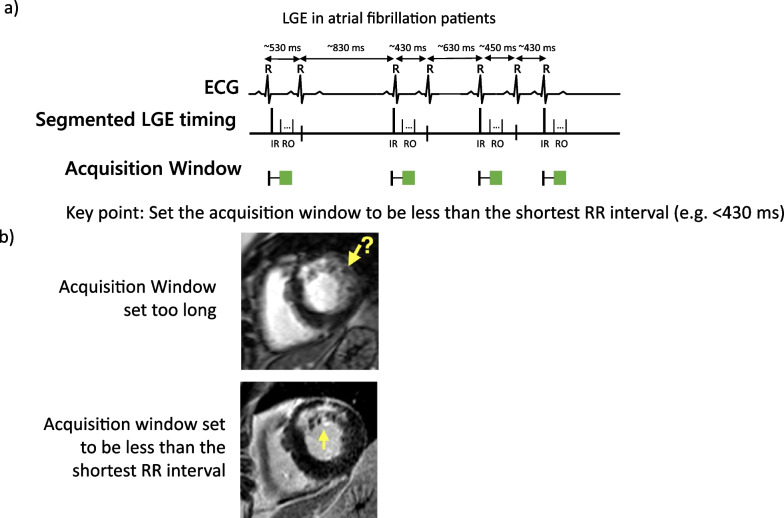
Adjustment to the data acquisition window for patients with atrial fibrillation. **a** In patients with atrial fibrillation (or other irregular rhythms), we adjust the data acquisition window such that the imaging readout is completed prior to the end of the shortest R–R interval between heart beats. **b** Examples of LGE images in a patient with atrial fibrillation. The top row shows in which the data acquisition window was set too long (> than the shortest R–R interval). There is substantial blurring of the subendocardium, and it is not clear if an infarct is present. The bottom row from the same patient in whom data acquisition was set to be less than the shortest R–R interval. Image sharpness is improved and the infarct is easily delineated from the blood pool and adjacent normal myocardium

Adjustments to the location of the acquisition window are typically required in patients with irregular arrhythmias, such as atrial fibrillation (AF). In patients with AF, the length of the cardiac cycle is variable, and when one compares one R–R interval to another, the main difference between heart beats is the duration of diastole (Fig. 6a). In other words, the duration of systole between heart rates in patients with AF is relatively similar, while the duration of diastole may be variable. Accordingly, at our center, we adjust the location of the data acquisition so that imaging occurs at the end of systole/early diastole. For most patients with AF, we adjust the timing such that the end of the image readout occurs prior to the end of the shortest R–R interval between heart beats (Fig. 6b). This strategy can be adapted for other irregular heart rhythms and is compatible with both single shot and segmented acquisitions.

#### Gating factor

**Fig. 7:**
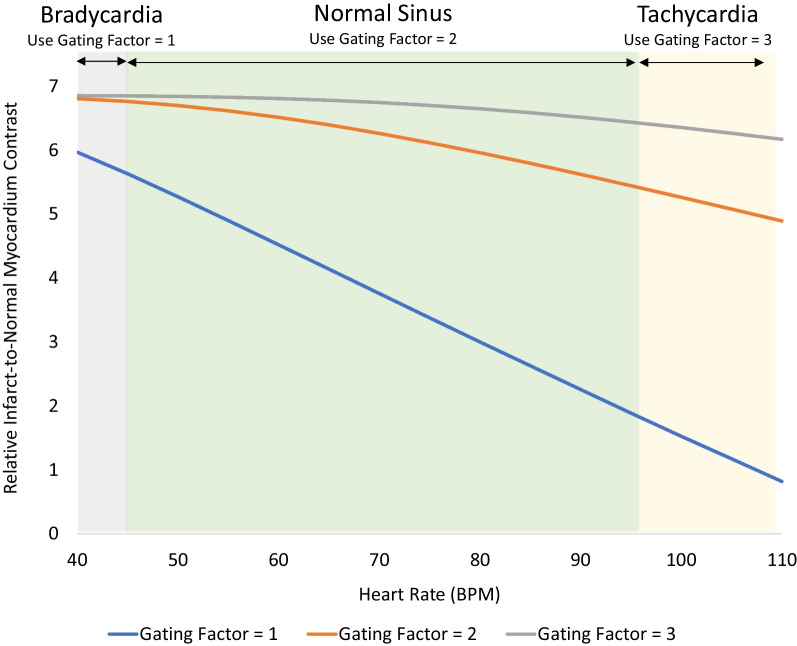
Simulation of relative image contrast for infarcted myocardium as a function of heart rate and gating factor. For patients with typical heart rates (45–100 bpm, green shading), a gating factor of 2 (orange line) is sufficient to allow for the majority of the longitudinal magnetization to recover between repeated inversion pulses with minimal saturation of magnetization. Thus, differences in image intensity between normal and infarcted myocardium are high and relatively flat across these heart rates with a gating factor of 2. For patients with tachycardia (> 100 bpm, yellow shading), a gating factor of 3 (gray line) is preferred since it maintains the time between repeated inversion pulses and allows longitudinal recovery to occur more fully. Compared to a gating factor of 2, the differences in image intensity between normal and infarcted myocardium are better maintained. The increase in total number of heart beats required to complete the image is partially offset by the increase in heart rate. For patients with significant bradycardia (< 45 bpm, gray shading), a gating factor of 1 (blue line) can be considered since the time between inversion pulses is longer and allows sufficient recovery between inversion pulses to occur. The decrease in number of heart beats required to complete the image reduces the breath hold time (assumes PSIR is not employed). Simulations using the Bloch equations assumed a GRE readout with 23 lines of k-space per heart beat, 19 degree readout flip angle, TI set to null normal myocardium, and an infarct T1 of 300 ms. PSIR = phase sensitive inversion recovery

Since successive IR preparation pulses are used to impart T1 weighting, wait beats are used to allow the recovery of longitudinal magnetization between pulses (Fig. 4). The gating factor controls the number of heart beats (or wait beats) between IR pulses. For patients with typical heart rates (~ > 45– < 100 bpm), a gating factor of 2 (one wait beat between IR pulses) is sufficient to allow for the majority of the longitudinal magnetization to recover (Fig. 7). However, in patients with tachycardia, a gating factor of 3 (2 wait beats) is preferable since the time between IR pulses is shorter and image intensity differences between enhanced and normal myocardium are better maintained. Conversely, in patients with significant bradycardia, a gating factor of 1 (0 wait beats) can be sufficient to achieve recovery of longitudinal magnetization.

#### Inversion time

The TI is defined as the time between the end of the non-selective inversion preparation to the center of k-space data acquisition (for a linear reordering, Fig. 4). Historically, the selection of TI for bright blood LGE has been one of the more difficult parameters for operators to learn to adjust accurately. Fortunately, the development of phase sensitive reconstruction (PSIR) [11], has greatly simplified the process. Using PSIR, operators are able to obtain high quality LGE images without the need for precise optimization of the TI. Nonetheless, we find that an understanding of how the TI is manually selected has clinical utility, and this knowledge can be used to identify specific pathologies, such as intracardiac thrombi or cardiac amyloidosis.

**Fig. 8:**
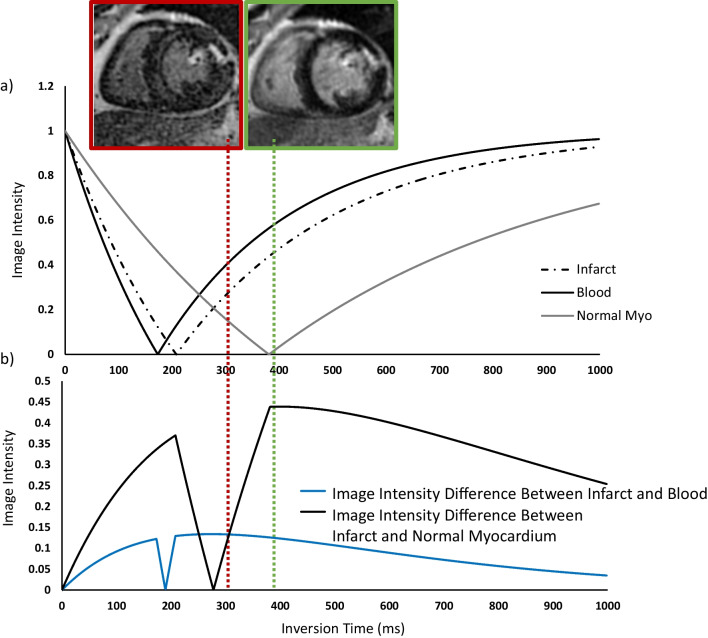
**a** Image intensity of normal and infarcted myocardium as a function of TI for magnitude reconstruction LGE. Simulations assumed a T1 of normal myocardium is 550 ms and infarcted myocardium is 350 ms, and blood is 300 ms. **b** Difference in image intensities between infarcted and normal myocardium as function of inversion time for magnitude reconstruction LGE. The time at which the magnetization of normal myocardium crosses zero is defined as the TI to null normal myocardium. This TI is considered ‘optimal’ because the image intensity difference between infarcted and normal myocardium is maximized (green box and line). Selection of a TI that is shorter than the optimal time (left of the green line) is problematic because the image intensity difference between infarct and normal myocardium can be significantly reduced (or possibly eliminated), and the relationship with the selected TI time is not linear. An indication that the TI is set ~ 20–50 ms too short is the presence of ‘etching’ (red box) which occurs at the interface between the blood pool and subendocardium due to the partial volume effect. The voxels situated at this interface include both myocardium (which is below the zero crossing) and blood pool (which is above the zero crossing), leading to a net magnetization of zero; these voxels appear black. The adjacent mid-myocardial voxels (below the zero crossing) and blood pool only voxels (above the zero crossing) are bright. Because of this artifact, we recommend that operators systematically select a TI that is slightly longer than optimal (~ 30 ms). The image intensity difference between infarct and normal myocardium with these slightly longer TIs (right of the green line) is predictable and stable. TI: inversion time

For manual selection of the TI, the goal is to select the TI so that the longitudinal magnetization of normal myocardium reaches just past the zero crossing (Fig. 8a), thereby ‘nulling’ normal myocardium. This TI also maximizes the difference in magnetization (and consequently, image intensity; Fig. 8b) between infarcted and normal myocardium.

Inaccurate selection of the TI may lead to missed detection of infarcts and incorrect infarct sizing, particularly if the TI selected is too short. In general, selection of TIs that are longer than the normal myocardium zero crossing maintains the difference in image intensities between normal and infarcted myocardium over a reasonable range. However, selection of TIs that are shorter than the normal myocardium zero crossing may create ambiguity in the relative image intensities of normal and infarcted myocardium (Fig. 8, red box). Specifically, when TIs that are somewhat shorter than the null time for normal myocardium are selected, differences in image intensity between normal and infarcted myocardium are variable or may even disappear. As a result, we train operators to select a slightly longer inversion time than the normal myocardium zero crossing in order to avoid acquiring images for which the TI may be too short ((Fig. 8, green box).

At our center, our operators are trained to manually adjust the TI by imaging iteratively with different inversion times. In general, a TI of 300 ms at 1.5 T or 400 ms at 3 T when using a linear K-space trajectory (50–100 ms shorter if centric) is a reasonable first estimate of the TI assuming a contrast dose 0.15 mmol/kg. One could consider reducing the initial TI estimate if a higher dose of contrast is given, imaging performed earlier after contrast administration, or the rate of contrast clearance is anticipated to be reduced.

**Fig. 9:**
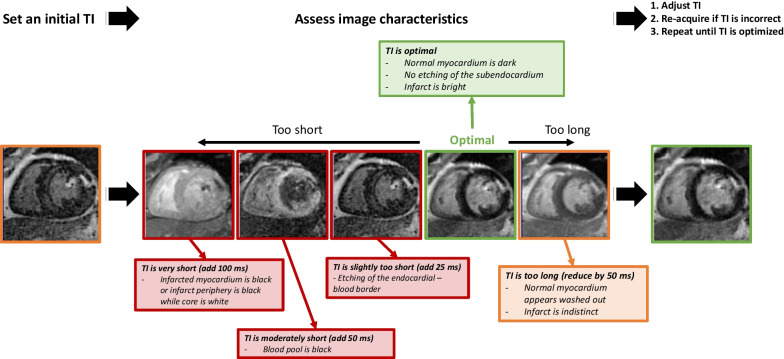
Iterative process for TI selection. After the acquisition of the initial LGE image, operators should adjust the TI based on the appearance of the myocardium. In particular, adjustments are imperative when the TI is set too short. Based on the imaging characteristics seen on the initial image, the TI should be adjusted (typically in small increments of 25–50 ms) accordingly. Using this iterative process, the optimal TI can usually be identified with 2–3 test images

The process for iteratively selecting the TI is shown in Fig. 9. In particular, we recommend that the operators learn to recognize when the TI is slightly too short. In this instance, myocardium will exhibit an ‘etched’ appearance due partial volume effects (see Fig. 8 legend). In most cases, the optimal TI can be identified after 1 or 2 “test” images. As the time after contrast injection increases, the optimal TI usually becomes longer. Thus, typically, the TI will need to be slowly incremented (e.g., 10–20 ms) to higher values based on the image appearance over the course of the examination.

A Look-Locker sequence or TI scout can also be used to provide an initial estimate of the TI [12]. However, due to readout effects and sampling interval errors, the Lock-Locker sequence only provides a reasonable first approximation of the optimal TI, usually underestimating the optimal TI by as much as 50 ms. Accordingly, additional manual iteration is still usually required to identify the optimal TI following the use of a Look-Locker sequence.

LGE with PSIR is an important method that reduces the complexity of TI selection [11]. The method allows the operator to select a “default” TI value to reconstruct images in which normal myocardium appears nulled by combining data from the conventional LGE acquisition with a second data acquisition (known as the ‘reference’). The “reference” data is acquired during the wait beat and is used for the phase sensitive reconstruction. The reference data are used to resolve the sign of longitudinal magnetization of a tissue species following the inversion preparation (e.g., used to determine if a tissue is at, above, or below zero/null point). Accordingly, following phase sensitive reconstruction, the image intensities on the PSIR image will be rendered based on relative T1. Tissue species with longest T1 (e.g., such as pericardial or pleural fluid) are displayed with the darkest image intensity. Viable myocardium has an intermediate relative T1 and is also dark but less dark than long T1 species. Tissue species with short T1 (e.g., myocardium with LGE or fat) will be displayed with the highest relative image intensity. Importantly, the differences in relative image intensity between normal myocardium and infarcted myocardium are maintained over a fairly wide range of TI times and obviates the problem of setting the TI too short.

As LGE imaging is typically acquired with a gating factor of two, the inclusion of PSIR comes at no cost in terms of breath-hold duration or SNR, and thus, our standard protocol includes PSIR. One caveat, however, is that a moderate degree of co-registration between the magnitude and reference data is required in order to avoid errors in the determination of phase for the PSIR image. Co-registration errors can occasionally occur in the PSIR image if large beat to beat changes in heart position occur due to respiratory motion or ectopy. In our experience, this is rarely an issue for breath held implementations as the amount of mis-registration is negligible. However, when using free breathing single shot implementations, mis-registration can be more common. The result can be inaccurate rendering of image intensity in the cardiac structures. Fortunately, in these instances, the magnitude reconstruction is still interpretable, provided that the TI was selected appropriately. For this reason, we teach operators to manually adjust the TI to null normal myocardium even when using PSIR, ensuring that both the magnitude and PSIR images are interpretable.

As noted earlier, one advantage of having a high level of proficiency in adjusting the TI enables operators to easily adapt bright blood LGE imaging to identify other pathologies, including intracardiac thrombi, MVO associated with acute MI, and cardiac amyloidosis. Typically, a change in approach to setting the TI can be used to highlight these pathologic findings.

**Fig. 10:**
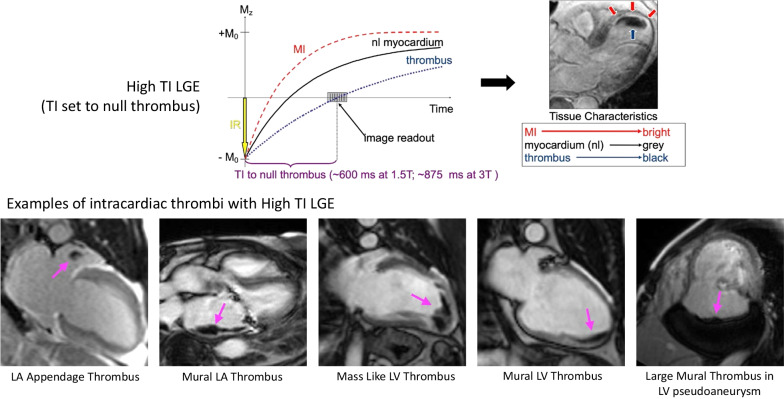
High TI LGE imaging for intracardiac thrombi. Top: Inversion recovery curves for myocardial infarction (MI), normal myocardium, and thrombus are shown. When a TI of 600 ms at 1.5 T or 875 ms at 3 T is used, intracardiac thrombi appear black, while other cardiac structures are gray or bright. Bottom: Examples of various intracardiac thrombi identified by high TI LGE are shown

Identification of intracardiac thrombi by LGE imaging exploits the principle that thrombi are generally avascular and do not display contrast enhancement [13]. In other words, while tissue species with perfusion take up contrast (leading to reduction in their T1), there is no significant change in the T1 of thrombus post-contrast. Consequently, the inversion time that nulls thrombus prior to contrast administration is the same TI post contrast. In our laboratory, this TI was empirically determined to be ~ 600 ms at 1.5 T and ~ 875 ms at 3 T (Fig. 10, top). Using this ‘high’ TI, thrombi will appear black, while other tissues with perfusion or blood will appear gray or bright (Fig. 10, bottom) on the LGE magnitude reconstruction. We find that this binary (e.g., black vs bright) image appearance is helpful in facilitating rapid identification of intracardiac thrombi. As a result, we perform high TI imaging for thrombi in all patients using the SSIR-bSSFP sequence variant in the short axis and 2-chamber image stacks shortly after contrast administration (see acquisition timeline in Fig. 2). Imaging in the 2-chamber orientation allows the visualization of the left atrial appendage in a longitudinal axis.

While thrombi can also be identified by standard LGE imaging by using PSIR (e.g., without selecting a high TI), the image appearance will not be binary. Instead, since tissue species are rendered according to their relative T1, fluid, thrombus, and viable myocardium will all appear dark. Specifically, fluid will have the most negative relative image intensity value (e.g., most dark), followed by thrombus, and then by viable myocardium (e.g., least dark). Adjusting the image window and level is often necessary to bring out differences in image intensities in these structures.

**Fig. 11:**
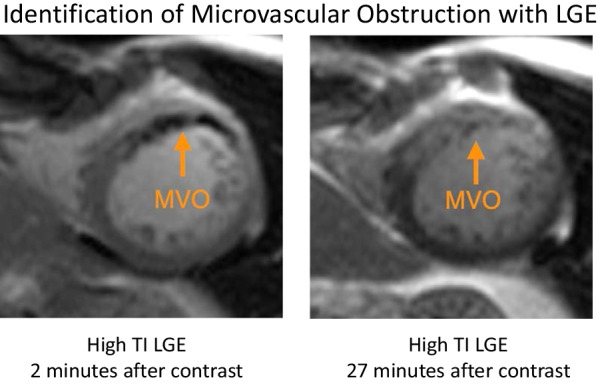
High TI LGE imaging of microvascular obstruction (MVO). Early after contrast administration, the central core of an infarct with MVO (left) appears black on high TI LGE. MVO (right) can be differentiated from thrombus by repeat imaging using the same sequence towards the end of examination. Unlike thrombus, infarcts with MVO will eventually exhibit contrast enhancement. MVO: microvascular obstruction

An added benefit of performing high TI SSIR-bSSFP imaging is that it enables the identification of MVO (“no-reflow”). The same high TI LGE technique used for thrombus imaging can also serve to identify acute infarcts with damaged microvasculature. In infarcts with MVO, there is minimal to no contrast enhancement early after contrast (e.g., the T1 in this region is long) and appear dark (hypointense). However, over the subsequent ~ 10–60 min, there is gradual contrast enhancement (via diffusion) beginning from the outer rim of infarct towards the central core (Fig. 11). The time course of contrast enhancement distinguishes MVO from thrombus, as the latter typically does not demonstrate appreciable contrast enhancement (with the possible exception of some chronic thrombi demonstrating an enhancing fibrotic cap). Thus, in patients with hypointense (dark) regions on early high-TI SSIR-SSFP, we repeat the sequence prior to ending the examination (typically ~ 20 min after contrast administration).

**Fig. 12:**
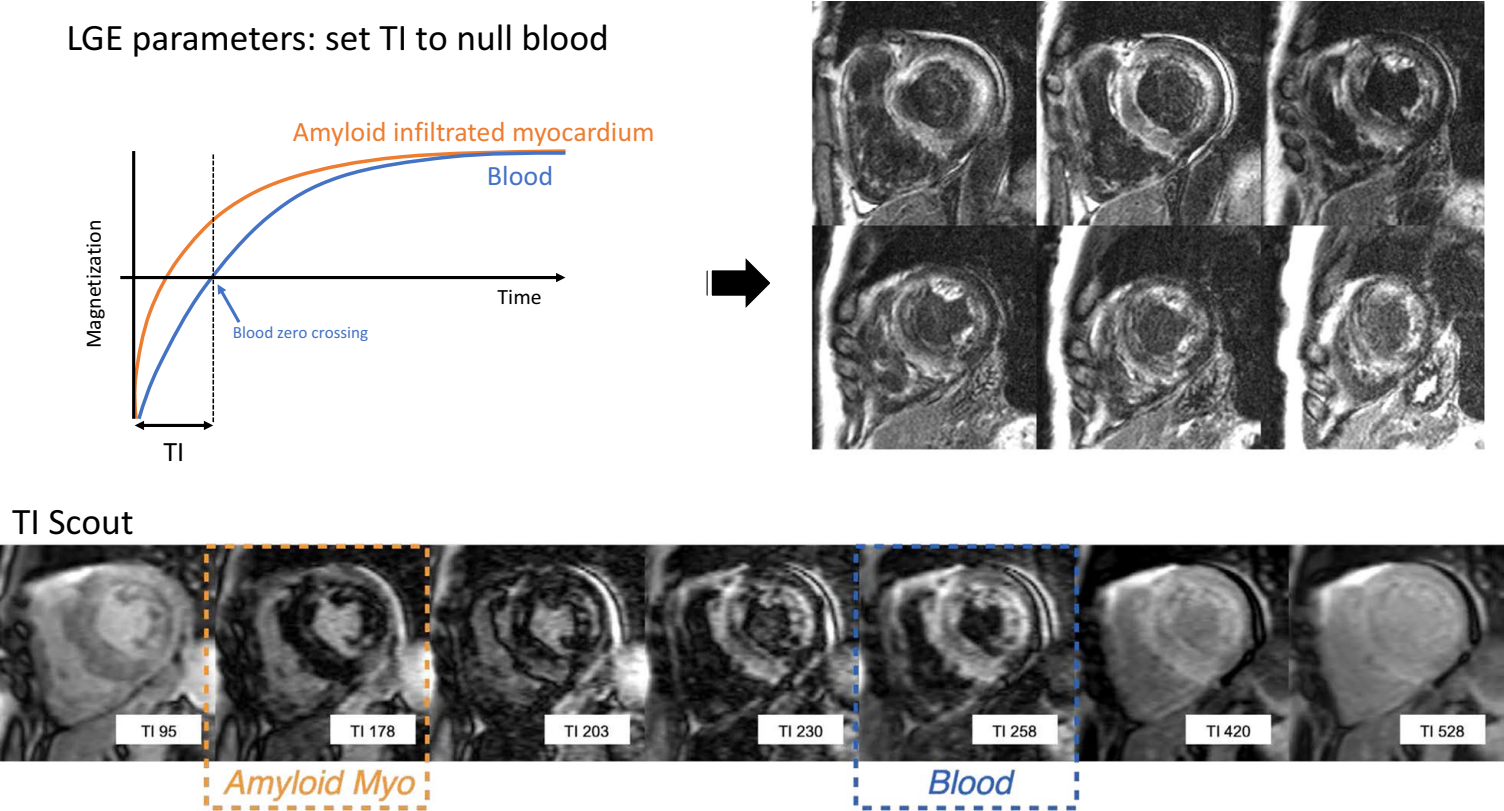
Cardiac amyloidosis. Top: Inversion recovery curves for the subset of patients with cardiac amyloidosis markedly altered gadolinium kinetics (left). Selection of the TI in this subset can often be confusing because, effectively, all the myocardium is abnormal and the null time for myocardium is shorter than blood. In this scenario, selecting an inversion time that null blood highlights the amyloid infiltration, and the regions with greater involvement have higher image intensity (right). Bottom: A TI scout can be used to quickly identify the presence of markedly altered gadolinium kinetics and to aid in the selection of the initial TI selection to null blood

Setting an appropriate TI in patients with cardiac amyloidosis may be challenging [14]. In our experience, we have found that this difficulty can be due to heterogeneity in contrast kinetics among patients with cardiac amyloidosis. In one subset of these patients, the contrast kinetics are markedly altered. The T1 of amyloid infiltrated myocardium is significantly shorter than blood T1, even early after contrast administration. In this circumstance, operators will find that they are unable to ‘null’ the myocardium using a typical TI, perhaps because the majority of the myocardium is abnormal and there is no ‘normal’ myocardium that can be nulled. Instead, we find that choosing an TI to null blood will highlight the extent of cardiac involvement (Fig. 12). In these patients, a TI scout can be used to quickly identify this alteration in contrast kinetics (e.g., the amyloid infiltrated myocardium is nulled at an TI that is shorter than blood) and to aid in selecting the appropriate TI to null blood (Fig. 11). In another subset of patients with cardiac amyloid, the contrast kinetics of amyloid infiltrated myocardium are conceptually similar to scar: the T1 of blood and amyloid infiltrated myocardium are both similarly short. In this case, the TI can be adjusted in the normal fashion to null normal myocardium.

## LGE in patients with cardiac devices

Performing CMR in patients with cardiac devices requires more complex screening procedures and changes to the imaging protocol in order to preserve safety and minimize image artifacts. In general, cardiac devices cause local image artifacts by disrupting the homogeneity of the magnetic field. For LGE, image artifacts are typically problematic when the device is large, (e.g., internal cardiac defibrillators) or in close proximity to the heart. The extent/size of the artifacts vary with the field strength (worse at 3 T than 1.5 T), readout type, and the use of conventional versus wideband inversion preparation pulses.

bSSFP pulse sequence variants are often hampered by the presence of off-resonance artifacts or large signal voids from the disruption of the magnetic field by the cardiac device. In comparison, GRE sequence variants tend to be resistant to off-resonance artifacts. However, in many cases, additional changes to a standard GRE implementation are still needed to reduce the impact of signal voids. For example, a typical implementation of GRE at 1.5 T (Table 1) may have large signal voids due to the rapid dephasing of spins near the device, and these artifacts often extend into the cardiac structures. Fortunately, the size of the signal void can be reduced by shortening the echo time, which is typically accomplished by increasing the receiver bandwidth (e.g., increasing to > 300 Hz/pixel at 1.5 T).

Off-resonance effects from an implanted device may also affect the ability of an inversion preparation to tip down spins. A device nearby the heart leads to a wider distribution of resonance frequencies within the heart (~ 2000–6000 Hz when a device is present vs 120 Hz when absent), which may exceed the spectral bandwidth of a conventional inversion pulse (~ 1000 Hz) [15–17]. Myocardium with resonance frequencies outside of the excitation bandwidth of the IR pulse are not inverted, thus creating an artifact that appears bright, irrespective of the presence of scar tissue.

Fortunately, in recent years, wideband (broadband) inversion pulses have become increasingly available to address this problem. A wideband inversion preparation has a larger spectral bandwidth (~ > 3000–4000 Hz) and is better able to tip down the full range of resonance frequencies within the heart when a device is present. Thus, normal myocardium can be uniformly nulled without the occurrence of bright artifacts due to this off-resonance effect. Although very useful in patients with cardiac devices, wideband inversions are not typically used in all patients because they require more power (B1) than conventional inversion pulses and have a lower inversion efficiency.

## Dark blood LGE

Several dark blood LGE sequences have been described in the literature [18]. In general, many of the pulse sequence parameters can be adjusted using the same guidelines as described above for bright blood LGE (SSIR-bSSFP and IR-GRE). However, since the mechanisms to generate image contrast between blood and infarcted myocardium are different, the process for selecting the optimal TI or other preparation time lengths to achieve dark blood conditions are sequence specific. A recent review describes the technical details of these sequences [18]. As discussed earlier, we use FIDDLE for dark blood LGE, and now use it in place of segmented IR-GRE in most patients.

**Fig. 13:**
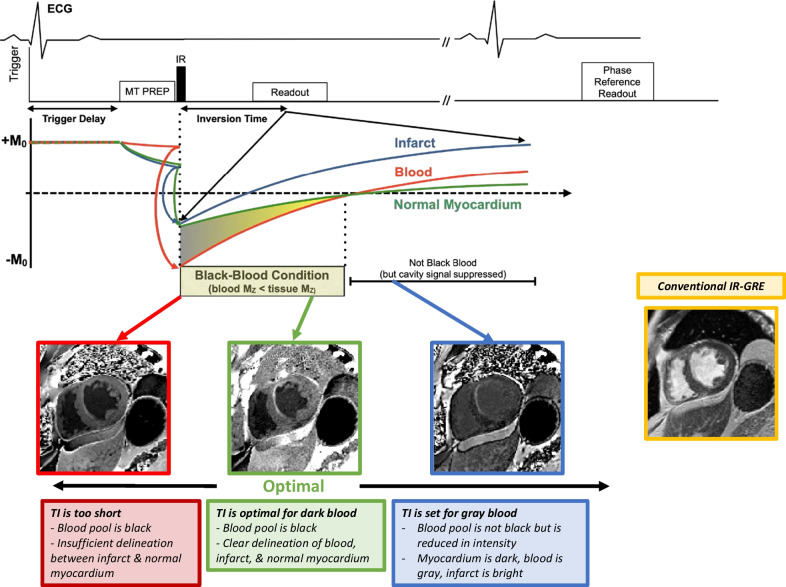
Top: FIDDLE pulse sequence timing diagram. The primary components are the: (1) preparatory module (MT prep), (2) inversion recovery pulse, (3) phase-sensitive reconstruction, and (4) inversion time, such that blood magnetization is less than tissue magnetization (Black-blood Condition). See text for details. MT = magnetization transfer. Bottom: The adjustment of inversion time is based upon image appearance after the acquisition of the first test image using a nominal TI (e.g., ~ 200 ms). Operators can identify the optimal dark blood TI by identifying the maximum inversion time that results in a dark blood image. Adapted from Kim et al. [6]

The typical pulse sequence parameters at 1.5 T and 3 T for segmented FIDDLE implementations used at our center are shown in Table 3. FIDDLE incorporates two preparation pulses with an bSSFP readout with phase sensitive reconstruction (Fig. 13) [6]. First, a magnetization transfer (MT) preparation is used to reduce the magnetization of blood more than that of myocardium. Next, an IR preparation then separates the blood, infarcted myocardium, and normal myocardium on the basis of T1. Finally, with appropriate selection of the TI and phase sensitive reconstruction, the resultant image provides separation of the different tissue species. The sequence was designed to be modular, and in principle, other magnetization preparation combinations that result in the same net magnetization could be incorporated into the sequence. However, in our experience, the MT followed by IR preparation results in the most uniform blood nulling and no issues with specific absorption rate at either 1.5 T or 3 T [6, 19].

**Table 2:** Typical parameters for single shot 2D IR-SSFP

Parameter	1.5 T	3.0 T
FOV	300–380 mm	300–380 mm
In-plane voxel size	1.4–1.8 × 2.0–2.5 cm	1.2–1.5 × 1.5–1.9 mm
Matrix size	208 × 150	256 × 200
Slice thickness	7 mm	7 mm
Flip angle	60°	45°
Segments	68	77
Inversion time (TI)	Variable	Variable
Bandwidth	601 Hz/pixel	977 Hz/pixel
TE (echo time)	1.24 ms	1.11 ms
TR (repetition time)	3.06 ms	2.64 ms
Gating factor	2	2
K-space ordering	Linear	Linear
Fat Sat	No	No
Asymmetric echo	Yes	Yes
Parallel imaging	2	2

We do not typically adjust the parameters of the MT preparation. Instead, the effect of the MT preparation on in-vivo tissue signal were measured empirically, and the signal behavior of FIDDLE was modeled to optimize the MT preparation flip angle, train length, and offset frequency [6]. As such, the MT parameters values are not typically changed (Table 3). Analogous to conventional bright blood LGE, however, we do manually adjust the FIDDLE inversion time (Fig. 13). We train the operators to pick a nominal TI (typically ~ 200 ms) and then based upon the image intensity of the blood pool, make an adjustment: if the blood pool is not black, the TI needs to be reduced; whereas, if the blood pool is black then the TI should be increased to the maximum value that still results in black blood. Usually, 1 or 2 test images are sufficient to find the optimal TI.

**Table 3:** Typical parameters for FIDDLE

Parameter	1.5 T	3.0 T
FOV	300–380	300–380
In-plane voxel size	1.1–1.7 × 1.1–1.7 cm	1.1–1.7 × 1.1–1.7 cm
Matrix size	256 × 205	256 × 205
Slice thickness	7 mm	7 mm
Flip angle	55°	44°
Segments	41	62
Inversion time (TI)	Variable	Variable
Bandwidth	514 Hz/px	930 Hz/px
TE (echo time)	1.92 ms	1.47 ms
TR (repetition time)	3.84 ms	2.94 ms
Gating factor	2	2
K-space ordering	Linear	Linear
Fat Sat	None	None
Asymmetric echo	Yes	Yes
Parallel imaging	2	2
MT pulses	19	19
MT flip angle	500°	400°
MT offset frequency	600 Hz	800 Hz

## Image interpretation for viability

Prior to interpretation, we adjust the image window and level of each image so that (a) noise remains detectable (e.g., nulled myocardium is not a single image intensity) and (b) regions with LGE are not saturated (e.g., LGE regions are not a single image intensity). For clinical reporting, we interpret LGE images using the 17-segment model recommended by the American Heart Association [20]. This model divides the basal and mid-cavities into six segments, and the apical cavity into four segments, with the true apex as one segment.

**Fig. 14:**
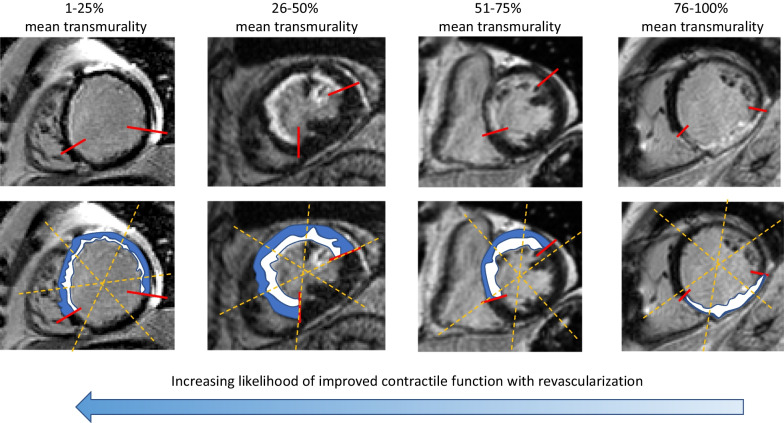
Representative LGE images with different mean transmural extent of infarction. Example images are shown on the top row, with a schematic highlighting the normal myocardium (blue) and the infarcted region (white). Dashed yellow lines represent the borders of the segment, and the red lines show the circumferential extent of the hyperenhancement. Importantly, interpretation should be based on the mean transmural extent of LGE within a segment, rather than the maximum transmural extent

The mean transmural extent of LGE within each segment is visually graded using a 5-point scale, where 0 = no hyperenhancement; 1 = hyperenhancement of 1–25% of the segment; 2 = hyperenhancement of 26–50% of the segment; 3 = hyperenhancement of 51–75% of the segment; and 4 = hyperenhancement of 76–100% of the segment [21]. We score the mean LGE transmural extent by visually comparing the region of LGE to the total wall thickness of the same segment where the LGE is present (LGE area/[LGE area + normal myocardium]) [22]. This approach to analysis is particularly crucial in patients with wall thinning, as using an adjacent or remote wall with normal wall thickness can lead to a systematic underestimation of viability. Representative examples are shown in Fig. 14.

In patients with coronary artery disease, the mean transmural extent of LGE is used to assess myocardial viability and to predict the likelihood of recovery of contractile function following coronary revascularization [21]. In myocardial segments with dysfunction, there is an inverse relationship between the mean transmural extent of LGE and the likelihood of improvement in contractility (Fig. 14). In other words, as the mean transmural extent of LGE decreases within a coronary perfusion territory, the likelihood of contractile function recovery increases if blood flow is restored.

## Conclusion

LGE imaging has become an integral component of the clinical CMR core cardiac examination with implementations on every CMR platform. As the use of LGE has expanded to patients with more severe illnesses, patient specific adaptations of the LGE sequence are often needed to maintain diagnostic image quality. Through the adjustments to the standard LGE protocol described in this document, image quality can be systematically improved even in the setting of high-volume clinical practice.

## Data Availability

Not applicable.

## Abbreviations

AF: Atrial fibrillation
bSSFP: Balanced steady state free precession
CMR: Cardiovascular magnetic resonance
ECG: Electrocardiogram
FIDDLE: Flow independent dark blood delayed enhancement
GBCA: Gadolinium based contrast agent
GRE: Gradient echo
IR: Inversion recovery
LGE: Late gadolinium enhancement
LV: Left ventricle/left ventricular
MI: Myocardial infarction
MT: Magnetization transfer
MVO: Microvascular obstruction
PSIR: Phase sensitive inversion recovery
SNR: Signal-to-noise ratio
SSIR: Single shot inversion recovery
TD: Delay time
TI: Inversion time
